# A commentary on “Peripheral nerve transfers for dysfunctions in central nervous system injuries: a systematic review”

**DOI:** 10.1097/JS9.0000000000003068

**Published:** 2025-07-14

**Authors:** Jianan Li, Keshuai Zhang, Wei Jiang

**Affiliations:** a Department of Neurosurgery, General Hospital of Northern Theater Command, Shenyang, China; b Department of Neurosurgery, Air Force Hospital of Northern Theater Command, Shenyang, China

*Dear Editor,*

We read with great interest the recent article titled “Peripheral nerve transfers for dysfunctions in central nervous system injuries: a systematic review”^[1]^ published in the *International Journal of Surgery*. The purpose of this study is to provide a systematic overview of recent developments in nerve transfers for dysfunctions that arise after CNS injury. The results showed that after CNS damage, nerve transfer typically enhances sensorimotor abilities in paralyzed limbs and bladder control. The technique effectively creates a “bypass” for signals and facilitates functional recovery by leveraging neural plasticity. However, a number of significant issues need to be addressed in future research.

First, a wide range of studies are included in the review, including human case reports and animal tests (in mice and monkeys), the majority of which are small series (single-center cases). This diversity makes it challenging to directly compare the results of different studies, particularly since the neuroplasticity mechanisms in animal models may differ significantly from those in humans. For example, rodents have a better neural regeneration ability than humans, and only a few of the 35 included studies are randomized controlled trials (RCTs), which limits the clinical applicability of the conclusions. Future research should focus on more standardized, large-scale clinical trials to verify the efficacy of neurotransplantation in treating human central nervous system (CNS) injuries and to clarify the boundaries between animal models and clinical applications^[2]^.

Second, the study lacks long-term follow-up data (e.g., over 5 years) and mostly describes short-term functional improvements following surgery (e.g., 6-12 months)^[3]^. For instance, the assessment of bladder function recovery only covers the effects from 8-12 months post-surgery, while the stability following nerve reinnervation and long-term complications (such as neuroma formation or functional decline) are not adequately addressed. Moreover, the improvement in motor function in patients with brain injury may vary over time, yet the review does not analyze the recovery trajectory at different time points. Future studies should incorporate long-term follow-up indicators, such as 10-year survival rates, functional maintenance, and changes in quality of life, to comprehensively evaluate the durability of the technology.

Third, although the analysis highlights the significance of postoperative rehabilitation, it does not go into great detail about particular rehabilitation regimens, including technical combinations, frequency, and intensity. For instance, the use of virtual reality (VR) and robot-assisted training is only briefly mentioned, without specifying how to tailor these protocols based on the type of nerve transfer (e.g., C7 transfer vs. lumbosacral nerve transfer). Furthermore, there is a lack of standardized parameters for non-invasive brain stimulation techniques, such as transcranial magnetic stimulation (TMS) and transcranial direct current stimulation (tDCS), which can affect the comparability of treatment outcomes. Future efforts should focus on developing phased, personalized rehabilitation guidelines and exploring the synergistic effects of multimodal rehabilitation.

This review systematically evaluated the application of neurotransplantation in CNS injury, and confirmed its potential for short-term functional improvement, but there are limitations such as sample heterogeneity, lack of long-term data, and insufficient standardization of rehabilitation. In the future, large sample RCTs, mechanism exploration and ethical standardization are needed to promote clinical transformation.All of this was noted in relation to the TITAN guideline, which calls for transparency in the reporting of artificial intelligence^[4]^.

## Data Availability

No data was used in this Letter to the Editor.
